# Tear Function and Abnormalities of Ocular Surface: Relationship with Subjective Symptoms of Dry Eye in Ibadan, Nigeria

**DOI:** 10.4103/0974-9233.53369

**Published:** 2008

**Authors:** CO Bekibele, AM Baiyeroju, AI Ajaiyeoba, EEU Akang, BGK Ajayi

**Affiliations:** * From The Department of Ophthalmology, University College Hospital and College of Medicine, University of Ibadan, Ibadan, Nigeria; ** From The Department of Pathology, University College Hospital and College of Medicine, University of Ibadan, Ibadan, Nigeria

**Keywords:** itching, pricking, dry eye, tear instability

## Abstract

**Objective:**

To determine the relationship between tear function and ocular surface abnormalities with subjective symptoms of dry eye.

**Methods:**

Patients with various ocular irritation symptoms suggestive of dry eye were examined for tear break up time (TBUT), Schirmer's test 1, and Rose Bengal staining pattern. They were compared with a group of asymptomatic healthy subjects.

**Results:**

There were 63 subjects, mean age 43.8 years (+/−14.7 years) with various complaints of dryness presenting as having irritation or foreign body sensation. A group of 17 asymptomatic subjects, mean age 42.1 years (+/−12.7 years) were studied as controls. There were 22 (34. 9 %) males and 41(65.1%) females in the symptomatic group while the control group had 4 (23.5%) males and 13(76.5%) females. Ocular irritations included itching 38(60.3%), pricking and itching 10 (15.9%). Mean Shirmer's test values were lower for symptomatic subjects (mean 14.5mm +/−12.3 right eye; 14.9mm +/−12.4 left eye), compared to the controls (23.0mm +/−13.4 right eye; 17. 9mm +/−13.4 left eye) P=0.02, for right eye and 0.4 for left. The mean TBUT were also lower amongst the symptomatic subjects (10.5 seconds, right eye and 10.1 seconds left eye), while for controls mean TBUT was 12.7 seconds right eye and 12.1 seconds left eye (P=0.2). Fifty –six out of 126 (46.8%) eyes of all symptomatic subjects compared to 7 out of 34 (20.6%) eyes of asymptomatic subjects had positive staining of conjunctiva with rose Bengal (p=0.06). For subjects with itching as the primary symptom 44.7% of them as opposed to 23.5% of the controls were likely to stain positive with rose Bengal, (sensitivity of itching as screening tool for dry eye was 81%, specificity 38.2%). Itching and pricking sensation together (sensitivity 46.2% and specificity improved to 65%). Rose Bengal grades were also inversely correlated with mean Schirmers values (Pearson correlation −0.429; P = 0.001) and TBUT (Pearson correlation -0.316, P=0.005).

**Conclusion:**

Itching and other ocular irritation symptoms may be indicative of underlying abnormal tear function and ocular surface damage, their presence calls for further examination for tear deficiency and prompt institution of treatment for dry eye.

Dry eye (keratoconjunctivitis sicca, tear instability) is a disorder of the tear film due to tear deficiency or excessive evaporation and is associated with symptoms of discomfort and ocular irritation such as feeling hot, dry, gritty or sandy, burning, smarting sensation, itching, watering or tearing.^1^,^2^ Dry eye is one of the commonest afflictions affecting humans, especially the aged. It has been observed that over half of the adult population and a large number of teenagers presenting in an eye clinic have symptoms related to ocular surface disease and dry eyes.^3^ Itching eyes may often be due to keratoconjunctivitis sicca (KCS) rather than allergy.^4^ Watering eyes may be caused by reflex tearing secondary to the irritation of KCS and ocular surface disease.^5^ Thus a high index of suspicion and use of diagnostic tests for dry eyes such as staining with Rose Bengal or Lissamine green dye would help in the diagnosis and thus institution of prompt treatment in many of these cases. Many patients with dry eyes however remain undiagnosed and untreated especially in underdeveloped countries where it may not be possible to procure appropriate ocular surface stains. Thus knowledge of symptoms and ocular surface changes and signs which correlate with appropriate stains would therefore help in early diagnosis and reduce patient suffering. A study from Florida, USA which examined the correlation between tear fluorescein clearance time and Schirmers test scores with ocular irritation symptoms observed decreased tear clearance time as a risk factor for ocular irritation, even in subjects with normal Schirmer scores.^6^ Another study which examined the distribution and association of dry eye symptoms, Schirmer test results, rose Bengal scores in a population based sample of 2240 Elderly Americans observed a high prevalence of symptoms of ocular irritation among the elderly, and there was minimal overlap between individuals identified by questionnaire, Schirmer test and rose Bengal scoring.^7^ A study which examined conjunctival cytokine expression in symptomatic moderate dry eye subjects observed that subjects with moderate dry eye had significantly higher symptom scores, higher tear osmolarity, shorter tear break up time than healthy controls^8^. There is paucity of information on the association between ocular irritation symptoms and tear dysfunction measurements from the underdeveloped countries such as Nigeria. The objective of the present study was to examine the relationship between tear function and ocular surface changes with ocular irritation symptoms suggestive of dry eye amongst subjects presenting to our institution.

## Materials and Methods

The study was carried out at the University College Hospital Ibadan between April 2002 and March 2005. Approval of the study was obtained from human ethics committee of University College Hospital, Ibadan. Ocular irritation was defined as patient's complaint of a feeling of discomfort from itching, pricking, burning, non specific irritation, dryness, and stinging of the eyes. Patients with complaints of ocular irritation not due to presence of foreign body or trichiasis or explained by obvious aetiology such as vernal conjunctivitis were included in the study. Asymptomatic patients with simple refractive errors but not on any ocular medications without diabetes, hypertension or other systemic disease, were examined as controls in the study. All subjects had routine eye examination with slit lamp, and an assessment of tear function using the Schirmer test, and the tear break up time. The conjuctival and corneal surfaces were stained by using fluorescein dye and later by rose Bengal stain.

Schirmer's test was done using 5mm by 35mm Whatmans filter paper with out prior instillation of topical anaesthetic drops, and the eye was gently dried with clean tissue paper. The filter paper was folded 5mm from one end and inserted midway between the outer and middle third of the lower lid. The patients were allowed to blink as necessary. The paper was removed after 5minutes and amount of wetting measured from the fold. A reading of less than 5mm was regarded as abnormal and was considered as indicative of dry eye.

Tear break up time (BUT) was measured after instilling fluorescein dye into the inferior conjunctival fornix of patients and allowing blinking several times before stopping. The tear film was examined with a broad beam of cobalt blue light for appearance of black spots or lines representing areas of dryness. The interval between the last blink and the appearance of first dry spot around the central cornea was the BUT. A BUT of less than 10sec was considered as abnormal.

Fluorescein dye staining using a strip instilled into the lower fornix was performed and the cornea surface examined for presence of punctate epithelial keratopathy, filaments, mucus plaques concretions. Rose Bengal staining was performed by using a one drop of 1 percent of Rose Bengal without prior instillation of topical anaesthetic. Intensity of staining was graded on a scale of 0-3 depending on the intensity (0=no stain, 1=mild, 2=moderate, 3=intense staining). Level of staining was determined 2minute after instilling the Rose Bengal stain. Grading of staining was done at the slit lamp for each of temporal and nasal conjunctiva as well as the cornea to make a maximum of 9 scores. A cumulative score of 3 was regarded as positive while a total score less than 3 was regarded as negative.^9^ Data was analysed by the use of SPSS computer software package.

## Results

A total of 80 subjects were screened and were found to be eligible for inclusion in the study. Sixty-three subjects, mean age 43.8 years (range 17-70 years), mean age 43.8 +/−14.7 years) presented with various complaints of ocular irritation. Seventeen asymptomatic subjects, mean age 42.1 years (range 22-64 years), served as controls. There were 22 (34. 9 percent) males and 41(65.1 percent) females in the symptomatic group while in the control group there were 4 (23.5 percent) males and 13(76.5 percent) females. Majority of those with ocular irritation had itching 38(60.3 percent), pricking and itching 10 (15.9 percent). The mean Schirmer's test values for symptomatic subjects were lower, 14.5mm +/−12.3mm right eye and 14.9 mm +/−12.4 mm left eye. For the control group the mean values were 23.0 mm +/−13.4 mm right eye, and 17. 9 mm +/−13.4 mm left eye. The difference in Schirmer's test values between symptomatic and controls were only statistically significant for the right eyes (P=0.02, t-test for equality of means). The mean tear break up time were also lower amongst the symptomatic subjects (10.5 seconds +/−5.5 seconds, right eye and 10.1 seconds +/−5.0 seconds left eye), while the control group had mean tear break up time of 12.7 seconds +/−5.1 seconds right eye and 12.1 seconds +/−5.3 seconds, left eye. The difference in the mean tear break up time between the symptomatic and asymptomatic groups were not statistically significant (P=0.2, t-test for equality of means). Fifty-six (46.8 percent) eyes of symptomatic subjects compared to 7 (20.6 percent) eyes of asymptomatic subjects had positive staining of conjunctiva with rose Bengal, suggesting ocular surface abnormality. The difference was however not statistically significant (P= 0.06). Rose Bengal grades were inversely correlated with mean Schirmer's readings (Pearson correlation −0.429, P < 0.05) and TBUT (Pearson correlation -0.316, P<0.05). Further evaluation of subjects who had itching as the primary symptom revealed that 44.7 percent of these patients compared to 23.5 percent of the controls were likely to stain positive with rose Bengal. The sensitivity of using itching alone for the diagnosis of ocular surface damage / dry eye was 81 percent and specificity 38.2 percent. With itching and pricking sensation together as main complaint, the sensitivity reduced to 46.2 percent but specificity improved to 65 percent. The main presenting symptoms and the corresponding test values are illustrated in Table 1.

**Table 1:** Mean Tear Function Test Values and Rose Bengal Staining in Subjects with Ocular Irritation Symptoms

Symptom	No	% Mean	Schirmer Test (mm)		Mean TBUT (sec)		Positive	Rose Bengal Stain
						
			RE	LE	RE	LE	No	%
Itching	38	60.3	14.2	14.7	11.2	10.6	17	44.7

Itching and Pricking	10	15.9	18.5	18.4	10.5	9.0	3	30.0

Itching and Any Other	4	6.4	15.0	13.5	6.7	7.0	3	62.5

Any Other	3	4.8	9.7	9.7	8.3	11.0	2	66.7

Pricking	2	3.2	12.5	20.5	13.5	12.5	1	50.0

Dryness	1	1.6	2.0	8.0	7.0	8.0	1	100.0

Pricking, Stinging and Itching	1	1.6	1.0	1.0	5.0	10.0	1	100.0

Pricking, Itching and Any Other	1	1.6	9.0	14.0	15.0	15.5	1	100.0

Stinging and Itching	1	1.6	5.0	1.0	5.0	5.0	1	100.0

Burning, Dryness, Stinging and Itching	1	1.6	25.0	20.0	10.0	10.0	0	0.0

Dryness and Itching	1	1.6	33	30	10.0	10.0	0	0.0

No Symptom	17	100.0	23.0	79	12.7	12.1	4	23.5

Any other = smarting, non specific irritation, peppery sensations

## Discussion

This study observed lower Schirmer's test values as well as tear break up time among patients with ocular irritation symptoms suggestive of dry eye than their healthy controls. The values were also inversely correlated with rose Bengal staining, suggesting that reduced tear wetting /tear instability of the ocular surface may have a role in the symptoms. Our finding is in keeping with previous studies which had also observed reduced schirmer's values and tear break up time among symptomatic dry eye patients.^2^,^10^,^11^ The importance of inadequate tear wetting on symptom generation is also confirmed by reduction in symptoms in patients diagnosed with dry eye and treated by punctal occlusion.^12^,^13^ The study also observed a relationship between ocular irritation symptoms and the presence of ocular surface abnormality as confirmed by positive rose Bengal staining in 46.8 percent of symptomatic subjects compared to only 23.5 percent of asymptomatic controls. Thus the presence of ocular irritating symptoms could be indicative of ocular surface damage in patients with tear instability. Ocular surface damage may result from increased tear osmolality due to excessive evaporation or reduced tear production or from inadequate mucin protection.^14^ Using itching alone as a screening tool for subjects with ocular surface abnormality these results produced a sensitivity of 81 percent and a specificity of 38 percent. This study implies that itching complaint could be a useful screening tool since a large percentage (81 percent) of those with surface abnormalities would be available for testing. However, the specificity of only 38 percent implies that many false positives would needlessly have been included because they had itching. With addition of pricking sensation to itching as the screening requirement, there was reduction in sensitivity to 46 percent but the specificity improved to 65 percent. Thus fewer false positives would be included but many more cases would be missed also. Unfortunately most of the other irritation symptoms studied did not have enough subjects available for adequate analysis but those available were observed to have their eyes stained by rose Bengal. It is recommended that all patients presenting with any ocular irritation whose cause is not clinically obvious to have a tear function evaluation as well as staining for ocular surface abnormality which may indicate an existing dry eye state. In a study to determine which subjective assessments and objective tests have clinical utility as diagnostic tools in ocular irritation associated with dry eye syndromes, Pflugfelder et al observed that symptoms were similar among all syndrome groups but were most severe in the Sjogren's group. Fluorescein tear break-up time was significantly faster in the aqueous tears deficiency (ATD) and meibomian gland disease (MGD) groups than that in controls. Schirmer scores were significantly lower in the ATD group than those in MGD and control groups. Schirmer scores also correlated inversely with rose Bengal staining. They concluded that subjective assessments and objective diagnostic tests have clinical utility as diagnostic tools in tear-film disorders.^15^

## Conclusion

Itching and other ocular irritation symptoms may be indicative of underlying abnormal tear function and ocular surface damage, their presence therefore calls for further examination for tear deficiency states and the prompt institution of treatment for dry eye syndromes.

## References

[1] McGill J, Liakos G, Seal D, Goulding N, Jacobs D (1983). Tear film changes in health and dry eye conditions. Trans Ophthal Soc UK.

[2] Afonso AA, Monroy D, Stern ME, Feuer WJ, Tseng SC, Pflugfelder SC (1999). Correlation of tear fluorescein clearance and Schirmer test scores with ocular irritation symptoms. Ophthalmol.

[3] Moss SE, Klein R, Klein BE (2004). Incidence of dry eye in an older population. Arch Ophthalmol.

[4] Fahim MM, Haji S, Koonapareddy CV, Fan VC, Asbell PA (2006). Fluorophotometry as a diagnostic tool for the evaluation of dry eye disease. BMC Ophthalmol.

[5] Gencoglu EA, Dursun D, Akova YA, Cengiz F, Yalcin H, Koyuncu A (2005). Tear clearance measurements in patients with dry eye syndrome using qualitative lacrimal scintigraphy. Ann Nucl Med.

[6] Pflugfelder SC, Tseng SC, Sanabria O, Kell H, Garcia CG, Felix C, Feuer W, Reis BL (1998). Evaluation of subjective assessments and objective diagnostic tests for diagnosing tear-film disorders known to cause ocular irritation. Cornea.

[7] Schein OD, Tielch JM, Munoz B, Bandeen-Roche K, West S (1997). Relation between signs and symptoms of dryeye in the elderly. A population-based perspective. Ophthalmol.

[8] Narayanan S, Miller WL, Mc Dermott AM (2006). Conjunctival cytokine expression in symptomatic moderate dry eye subjects. Invest Ophthalmol Vis Sci.

[9] Bron AJ, Mengher LS (1989). The ocular surface in keratoconjunctivitis sicca. Eye.

[10] Xu KP, Yagi Y, Tsubota K (1996). Decrease in corneal sensitivity and change in tear function in dry eye. Cornea.

[11] Khurana AK, Chaudhary R, Ahluwalia BK, Gupta S (1991). Tear film profile in dry eye. Acta Ophthalmol (Copenh).

[12] Dursn D, Ertan A, Bilezikci B, Akova YA, Petit A (2003). Ocular surface changes in keratoconjunctivitis sicca with silicone punctual plug occlusion. Curr Eye Res.

[13] Horwath-Winter J, Berghold, A, Schmut O, Floegel I, Solhdju V, Bodner E, Schwantzer G (2003). Evaluation of the Clinical Course of Dry Eye. Arch Ophthalmol.

[14] Xu KP, Yagi Y, Tsubota K (1996). Decrease in corneal sensitivity and change in tear function in dry eye. Cornea.

[15] Pflugfelder SC, Tseng SC, Sanabria O, Kell H, Garcia CG, Felix C, Feuer W, Reis BL (1998). Evaluation of subjective assessments and objective diagnostic tests for diagnosing tear-film disorders known to cause ocular irritation. Cornea.

